# Blue light as an eco-elicitor effectively enhances flavonoid biosynthesis and antioxidant capacity in dandelion revealed by integrated metabolomic and transcriptomic analyses

**DOI:** 10.1186/s12870-026-08137-1

**Published:** 2026-01-19

**Authors:** Qiaojuan Xing, Yao Wang, Xuankai Zhou, Xuerui Cai, Shihong Zhang

**Affiliations:** 1 College of Plant Protection, Shenyang Agricultural University, Shenyang, 110866 China; 2 College of Horticulture, Shenyang Agricultural University, Shenyang, 110866 China; 3 The Key Laboratory for Extreme-Environmental Microbiology, College of Plant Protection, Shenyang Agricultural University, Shenyang, 110866 China

**Keywords:** Dandelion, Light, WRKY, Flavonoid, Genome-wide identification

## Abstract

**Background:**

Dandelion (*Taraxacum mongolicum*) is a globally important medicinal herb rich in antioxidant flavonoids. Blue light (BL) serves as a key environmental signal known to regulate the biosynthesis of plant secondary metabolites. However, the molecular mechanisms in regulating flavonoid biosynthesis in non-model medicinal plants like dandelion remain poorly understood, particularly the roles of WRKY transcription factors (TFs) in this process.

**Results:**

BL irradiation not only inhibited vegetative expansion but also enhanced photosynthetic pigment accumulation in dandelion. This treatment further boosted the plant’s antioxidant capacity and elevated its total flavonoid content. Notably, the levels of key antioxidant flavonoids such as rutin, apigenin, and quercetin derivatives were significantly increased. Transcriptomic profiling indicated a coordinated upregulation of structural genes (including *CHS*, *CHI*, *ANR*, *DFR*, *UGT*) in the flavonoid biosynthesis pathway. Genome-wide analysis identified 69 WRKY TFs (TmWRKYs) in dandelion. Among them, *TmWRKY2*, *TmWRKY 21*, *TmWRKY 47*, and *TmWRKY 55* were significantly induced by BL. Correlation analyses indicated that *TmWRKY2* and *TmWRKY55* expression was strongly positively correlated with both the expression of key flavonoid biosynthetic genes and the accumulation of antioxidant flavonoids. Furthermore, promoter analysis of these correlated structural genes revealed abundant W-box cis-elements, suggesting potential direct regulation by these WRKY TFs.

**Conclusions:**

Our study suggests that BL enhances flavonoid accumulation in dandelion, likely through a transcriptional activation model​ involving specific WRKY TFs, including TmWRKY2 and TmWRKY55. The identification of W-box elements in the promoters of key structural genes further supports this proposed regulatory network. These findings provide novel insights into light-transcription factor-metabolite interactions in medicinal plants and highlight potential targets for molecular breeding and cultivation strategies to improve dandelion’s nutraceutical value.

**Supplementary Information:**

The online version contains supplementary material available at 10.1186/s12870-026-08137-1.

## Introduction

Dandelion (*Taraxacum mongolicum*), a perennial herb in the Asteraceae family, is widely distributed across the Northern Hemisphere [1] and renowned as the “Queen of Herbs” due to its diverse medicinal and nutritional properties [2–6]. It is extensively applied in traditional Chinese medicine [7], functional foods, nutraceuticals [8], daily chemical products [9], and feed additives [10, 11]. Most of these applications is largely attributed to its potent antioxidant properties. Owing to its abundance in natural resources and rich content of diverse bioactive compounds-especially flavonoids, a special class of natural antioxidants-dandelion has garnered significant attention [12–14]. Notably, oxidative stress caused by excessive free radicals is implicated in various diseases and aging processes [15, 16]. Therefore, enhancing antioxidant activity is considered a crucial strategy for managing such oxidative stress-related diseases. Dandelion flavonoids, including luteolin, quercetin, apigenin, and rutin, counteract free radicals by serving as electron donors, which is a key mechanism underlying their significant antioxidant activity [12, 17].

The biosynthesis of antioxidant flavonoids is dynamically regulated by various environmental factors. Among them, light quality, particularly red light (RL, 600–700 nm) and blue light (BL, 400–500 nm), serves as a pivotal regulatory signal [18, 19]. When plants are exposed to RL, photoreceptors, primarily phytochromes, are activated, and these signals are transmitted to the nucleus, triggering the upregulation of critical enzymes in the phenylpropanoid pathway, such as phenylalanine ammonia-lyase (PAL) and chalcone synthase (CHS), and subsequently promoting the synthesis of antioxidant compounds such as anthocyanins, flavonoids, and phenolics [20–22]. Similarly, BL exposure enhances the accumulation of total flavonoids and phenolic compounds in basil [23], mugwort [24], sweet pepper [25], rhodiola [26], and cabbage [27], concomitantly increasing their antioxidant capacity. Mechanistically, this enhancement is attributed to the BL-induced transcriptional activation of key biosynthetic genes. For instance, in rice, BL exposure increases the expression of genes including PAL, 4CL, CHS, CHI, F3H, and FLS at both the transcriptional and translational levels [28]. Therefore, both RL and BL act as pivotal environmental modulators of flavonoid biosynthesis, primarily through the photoreceptor-mediated transcriptional activation of structural genes in the phenylpropanoid pathway.

The precise transcriptional activation of flavonoid pathway genes by light is ultimately orchestrated by specific TFs. Several TF families, including bZIP [29], BBX [30], ERF [31], are implicated in this light-induced process. Notably, the WRKY TF family has garnered increasing attention for its potential role in mediating plant responses to environmental cues, including light signaling, and in regulating secondary metabolism​ [32]. WRKY TFs are characterized by the conserved N-terminal WRKYGQK motif and C-terminal zinc finger domains (C2H2 or C2HC) [33, 34]. They are classified into Groups I (two WRKY domains and C2H2), II (single WRKY domain and C2H2, with subgroups IIa-IIe), and III (single WRKY domain and C2HC), and regulate downstream targets by binding to W-box cis-elements (TTGACC/T) in promoters, influencing diverse processes including secondary metabolism [34–38]. Although some WRKYs have been shown to directly activate flavonoid biosynthetic genes in other species [39–41], a comprehensive genome-wide analysis of the WRKY family in dandelion is lacking, and its potential role in mediating the specific effects of BL on flavonoid biosynthesis remains entirely unexplored.

In this study, we demonstrated that BL irradiation enhances the antioxidant capacity of dandelion by promoting flavonoid biosynthesis. To explore the molecular mechanism underlying this process, we employed an integrated metabolomic and transcriptomic analysis. This approach allowed us to identify candidate BL-responsive WRKY TFs that potentially regulate flavonoid accumulation. Our study provides novel insights into the light-mediated regulatory network of flavonoid biosynthesis in dandelion and offers valuable genetic resources for strategies aimed at improving its medicinal quality.

## Results

### Effects of light quality on aboveground morphology, biomass, and photosynthetic pigments in dandelion

**Fig. 1:**
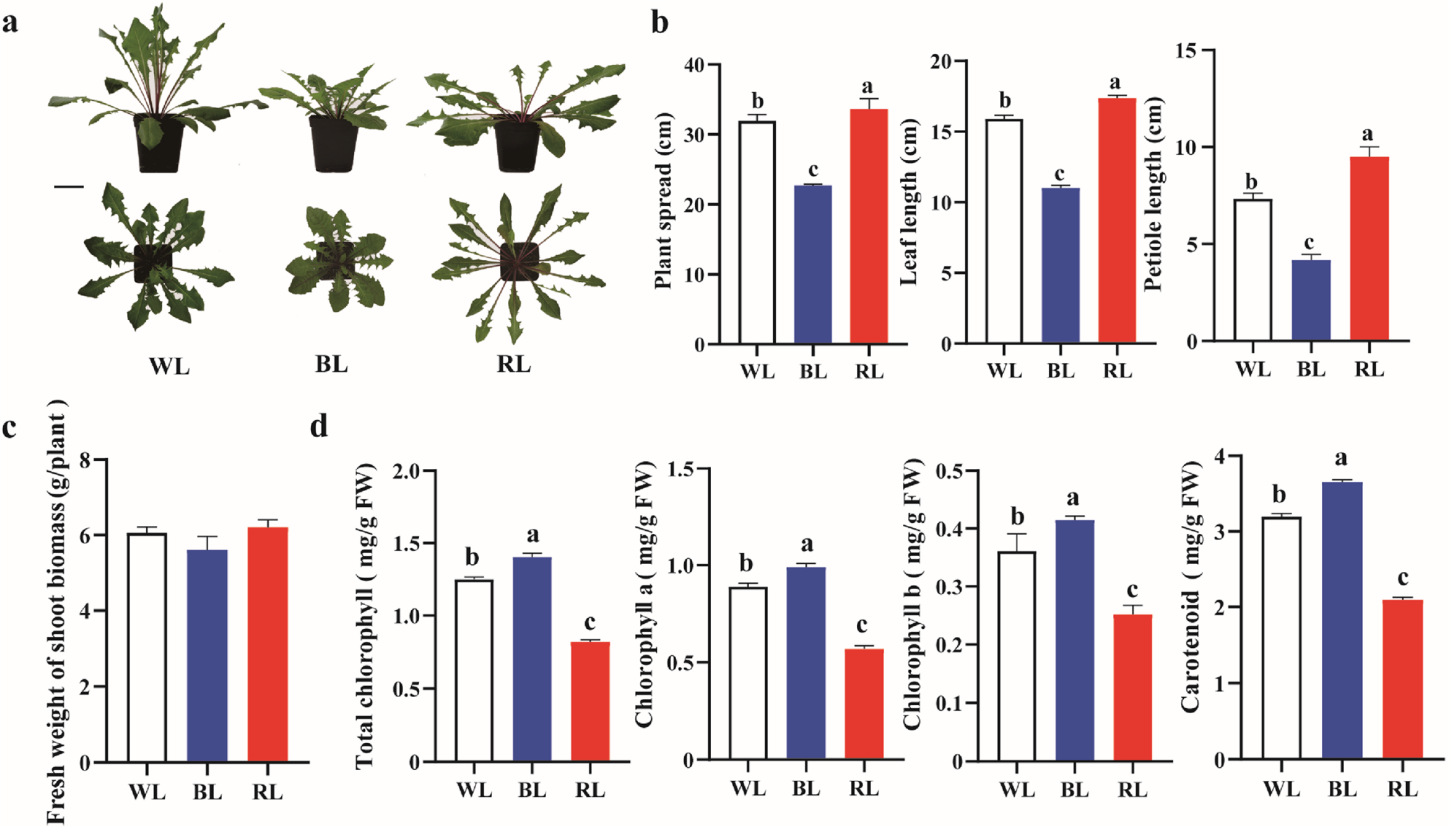
Morphological, biomass, and photosynthetic pigment responses of dandelion to different light quality treatments.​ **a** Representative plant phenotype. **b** Morphological parameters, including plant spread, leaf length, and petiole length. **c** Fresh shoot biomass. **d** Contents of photosynthetic pigments (chlorophyll *a*, chlorophyll *b*, and carotenoids). Data are presented as mean ± SD (*n* = 3). Different lowercase letters above the bars indicate significant differences (*P* < 0.05; one-way ANOVA) among treatments. The scale bar in (a) represents 5 cm. WL, white light; BL, blue light; RL, red light

The growth and development of the aboveground parts in dandelion were significantly influenced by the light quality treatments. Compared with the WL and RL treatments, BL irradiation results in a more compact morphology. This was quantitatively demonstrated by a significant decrease in plant spread (by approximately 19.9% and 32.5%, respectively), leaf length (by approximately 30.8% and 36.7%, respectively), and petiole length (by approximately 42.9% and 56.11%, respectively) (Fig. 1a, b). Despite this pronounced effect on morphology, no significant difference was observed in the fresh shoot biomass of dandelion among the three light quality treatments (Fig. 1c). In contrast, BL significantly promoted the biosynthesis of photosynthetic pigments. The contents of chlorophyll a, chlorophyll b, total chlorophyll, and carotenoids in BL-treated leaves were significantly the highest, with total chlorophyll approximately 12.4% and 71.1% higher than in WL and RL, respectively (Fig. 1d), while the RL group showed the lowest pigment content. Together, these results indicate that BL effectively suppresses the expansive growth​ of vegetative organs like leaves and petioles, while simultaneously and potently promoting the accumulation​ of photosynthetic pigments.

### Blue light enhances antioxidant capacity and total flavonoid content

**Fig. 2:**
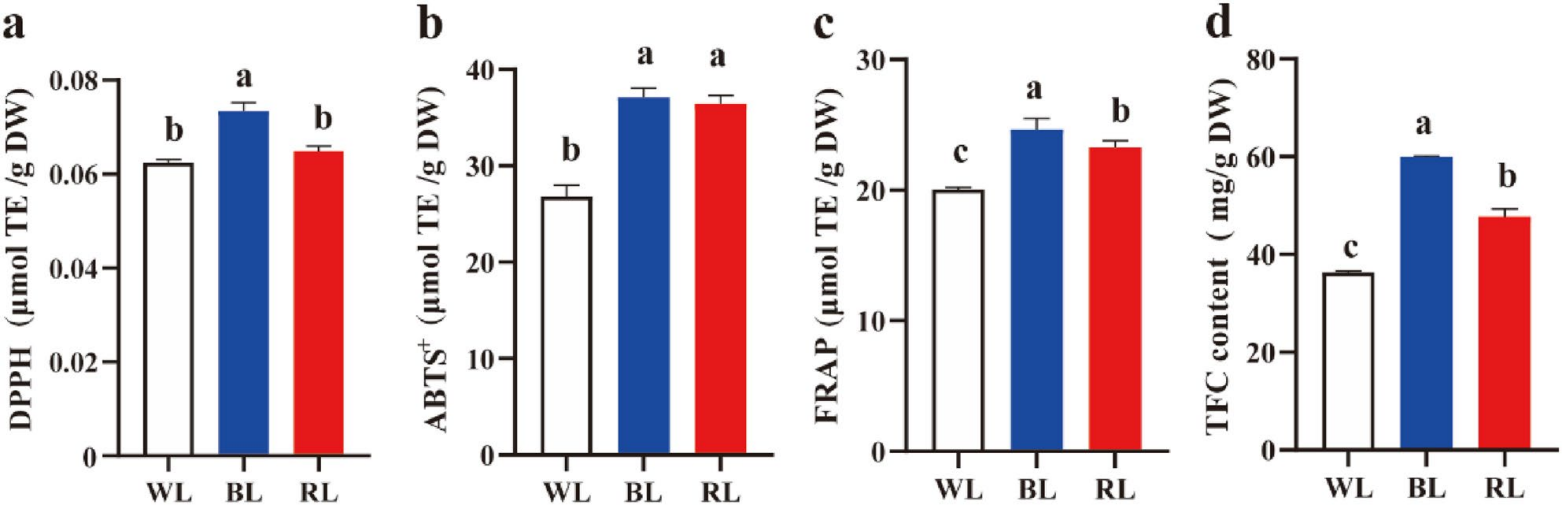
Effects of light quality on the antioxidant capacity and total flavonoid content (TFC) in dandelion. **a** DPPH scavenging activity. **b** ABTS^+^ radical scavenging activity. **c** FRAP assays. **d** TFC content. Data are presented as mean ± SD (*n* = 3). Different lowercase letters above the bars indicate significant differences (*P* < 0.05; one-way ANOVA) among treatments. WL, white light; BL, blue light; RL, red light

To investigate the effects of light quality on the antioxidant properties of dandelion, we assessed scavenging activities (DPPH and ABTS^+^) and ferric reducing antioxidant power (FRAP) activities, as well as determined the total flavonoid content (TFC). The results showed that DPPH, ABTS^+^, and FRAP were all markedly higher in the BL treatment compared to the WL and RL treatments (Fig. 2a-c). Specifically, the DPPH scavenging activity under BL was approximately 17.5% and 13.1% higher than that under WL and RL, respectively. Similarly, the ABTS⁺ scavenging activity and FRAP value under BL were significantly increased by about 38.3% and 22.9% compared to WL. The lowest antioxidant activity was consistently observed under WL. In addition, consistent with the enhancement in antioxidant capacity, TFC accumulation was also most pronounced under BL, showing a 64.7% and 25.4% increase compared to WL and RL conditions​ (Fig. 2d). These findings suggest that BL may effectively enhance the antioxidant capacity of dandelion by promoting flavonoid biosynthesis.

### Analysis of metabolic differences in dandelion under different lighting conditions

**Fig. 3:**
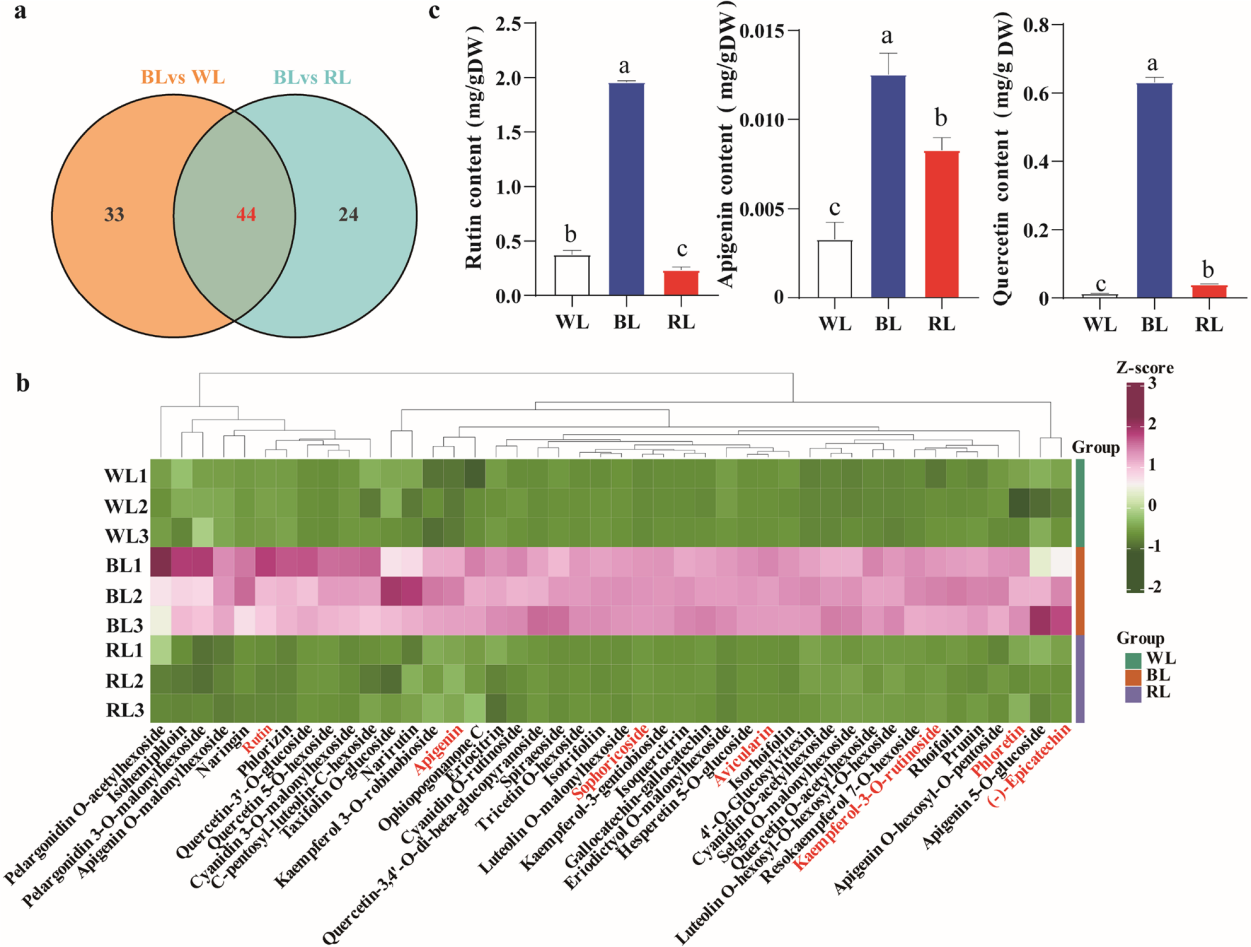
Differential metabolite profiling in dandelion leaves under different light treatments. **a** Venn diagram of the DAMs for the different comparisons. **b** Heatmap of differential flavonoid accumulation patterns. **c** Quantification of quercetin, apigenin, and rutin. Values represent mean ± SD (*n* = 3). Different lowercase letters above the bars indicate significant differences (*P* < 0.05; one-way ANOVA) among treatments. WL, white light; BL, blue light; RL, red light

We assessed light quality effects on dandelion metabolism using LC-MS/MS. Principal component analysis (PCA) revealed a clear separation among the WL, RL, and BL treatments along the first two principal components, which collectively accounted for 70.25% of the total variance (PC1: 39.41%; PC2: 30.84%) (Supplementary Fig. 1a). The three biological replicates for each light condition clustered tightly together (Supplementary Fig. 1a). By analyzing the correlations between samples, we observed that biological replicates within each group showed high consistency. A higher correlation coefficient within groups and a lower correlation between groups generally indicate more reliable identification of differential metabolites. Pearson’s correlation coefficient was used to evaluate the reproducibility of biological replicates. The sample correlation clustering results showed significant differences between groups and minimal variation within groups, indicating good reproducibility among the test samples (Supplementary Fig. 1b). Among 1,053 identified metabolites across nine samples, flavonoids constituted a major class (157 metabolites, Supplementary Fig. 1c). Differentially accumulated metabolites (DAMs) were identified based on the following thresholds: variable importance in the projection (VIP) ≥ 1 combined with fold change (FC) ≥ 1.5 or ≤ 0.667 at the P-value < 0.05 level. The KEGG analysis revealed that DAMs in the BL group were significantly enriched in “phenylpropanoid biosynthesis”, “flavonoid biosynthesis”, and “flavone and flavonol biosynthesis” pathways compared to the WL and RL groups (Supplementary Fig. 1d, e), indicating that BL may specifically activate the phenylpropanoid-flavonoid biosynthetic pathway, leading to a more pronounced accumulation of associated metabolites. Furthermore, comparative analyses of “BL vs WL” and “BL vs RL” identified 77 and 68 DAMs, respectively (Fig. 3a). Among these, 44 metabolites overlapped between the two comparisons (Fig. 3a; Supplementary Table 1). Notably, seven overlapping metabolites-avicularin, rutin, sophoricoside, (-)-epicatechin, kaempferol-3-O-rutinoside, apigenin, and phloretin-exhibited significantly higher accumulation under BL, showing fold increases of approximately 1107.1 and 391.9 (avicularin), 17.6 and 6.5 (rutin), 10.8 and 25.6 (sophoricoside), 2.9 and 3.1 ((-)-epicatechin), 2.3 and 2.6 (kaempferol-3-O-rutinoside), 2.2 and 3.3 (apigenin), and 1.7 and 1.9 (phloretin) compared to RL and WL, respectively (Supplementary Table 1). All these compounds have been documented in dandelion and possess documented​ antioxidant properties [14] (Fig. 3b). Structurally, both avicularin and rutin are quercetin derivatives, underscoring close metabolic relationships within the flavonoid pathway. Importantly, rutin, apigenin, and its parent flavonol quercetin are recognized as key bioactive constituents of dandelion. HPLC quantification validated these metabolomic trends, demonstrating significant increases induced by blue light treatment (Fig. 3c).​ Taken together, these results demonstrate that BL may specifically induce the phenylpropanoid-flavonoid biosynthetic pathway in dandelion, thereby promoting the accumulation of key antioxidant flavonoids.

### Transcriptome analysis of dandelion under different lighting conditions

To elucidate the transcriptional regulation of BL-induced flavonoid biosynthesis in dandelion, we conducted comparative transcriptome profiling of leaves under WL, RL, and BL with three biological replicates per condition (nine samples total). PCA showed a clear separation of the transcriptomes by treatment along the first two principal components (PC1:28.1%, PC2:24.04%; cumulative variance:52.14%) (Supplementary Fig. 2a). We identified 3,480 and 1,727 differentially expressed genes (DEGs; FDR < 0.05, |log2Fold Change|≥1) in the comparisons of “BL vs RL” and “BL vs WL”, respectively. Among these, 675 DEGs were shared between the two comparisons (Supplementary Fig. 2b). KEGG enrichment analysis of these 675 common DEGs revealed significant enrichment​ in metabolic pathways, with a strong emphasis on flavonoid metabolism, including “Flavonoid biosynthesis”, “Flavone and flavonol biosynthesis”, and “anthocyanin biosynthesis” (Supplementary Fig. 2c). These results suggest that BL may specifically activate the transcription of the flavonoid biosynthesis pathway.

**Fig. 4:**
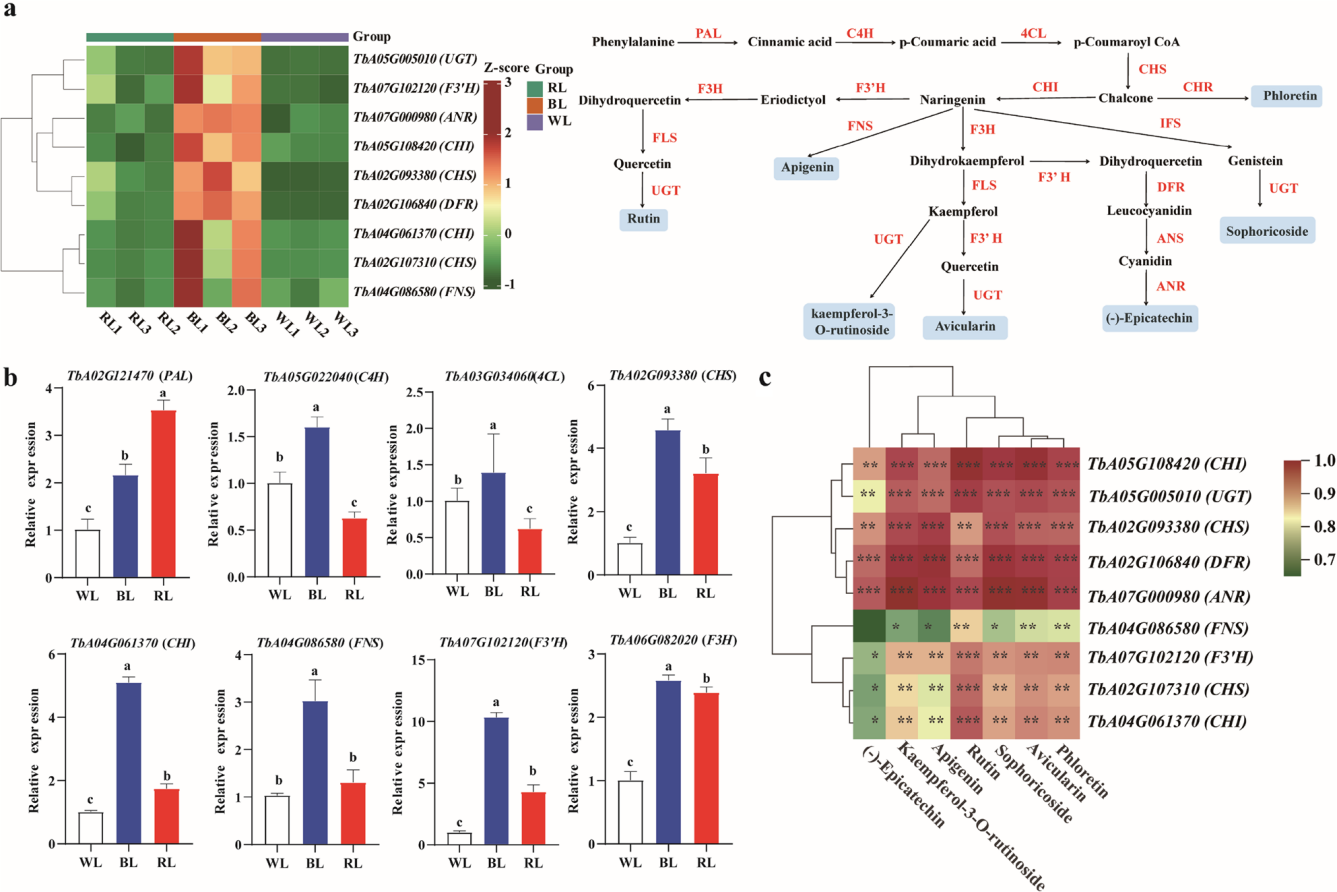
Biosynthesis of flavonoids in dandelion leaves under different light treatments. **a** Simplified diagram showing the flavonoid biosynthetic pathways and heatmaps of the relative expression of genes encoding enzymes involved in flavonoid synthesis under different light treatments. Phenylalanine ammonia-lyase (PAL), cinnamate 4-hydroxylase (C4H), 4-coumarate CoA ligase (4CL), chalcone synthase (CHS), chalcone isomerase (CHI), flavanone 3-hydroxylase (F3H), flavonoid 3′-hydroxylase (F3′H), flavonol synthase (FLS), flavone synthase (FNS), chalcone reductase (CHR), isoflavone synthase (IFS), dihydroflavonol 4-reductase (DFR), anthocyanidin synthase (ANS), anthocyanidin reductase (ANR), and flavonoid 3-O-glucosyltransferase (UGT) were identified. Seven antioxidant flavonoids are shown in light blue rectangles; the red text on the arrows indicates the enzymes catalyzing the corresponding reactions, and the arrow directions represent the biosynthetic pathway. **b** qRT-PCR validation of DEGs in the flavonoid biosynthesis pathway in dandelion leaves under different light treatments. Data are presented as mean ± SD (*n* = 3). Different lowercase letters above the bars indicate significant differences (*P* < 0.05; one-way ANOVA) among treatments. c Correlation analysis of seven antioxidant flavonoids and flavonoid biosynthesis genes in dandelion under a threshold of 0.8 was performed. _*_ Indicates significant differences at the *P* < 0.05 level; _**_ indicate significant differences at the *P* < 0.01 level; _***_ indicate significant differences at the *P* < 0.001 level. WL, white light; BL, blue light; RL, red light

Further analysis of the flavonoid biosynthetic pathway identified 29 key enzyme-encoding genes from the transcriptome database (Supplementary Table 2). This gene set included genes acting from the initial steps of the phenylpropanoid pathway *(PAL*, *C4H*, *4CL*) to later steps specific to flavonoid formation (*CHS*, *CHI*, *F3’H*, *DFR*, *ANR*, *UGT*). Among these, 9 genes showed significantly higher expression under BL than under both WL and RL (Fig. 4a). The accuracy of the RNA-seq data was confirmed by qRT-PCR analysis of eight selected genes, the results of which were entirely consistent with the transcriptomic trends (Fig. 4b). Furthermore, correlation analysis showed that these nine genes exhibited strong correlations (|r| > 0.8; *P* < 0.01, < 0.05, or < 0.001) with the seven antioxidant flavonoids (Fig. 4c). These data collectively suggest that BL may specifically induce flavonoid biosynthesis by coordinately regulating the upregulation of key structural genes throughout the phenylalanine-flavonoid pathway.

### Genome-wide identification and classification of TmWRKYs

Previous studies have reported that WRKY TFs are widely involved in flavonoid biosynthesis in plants [32–34]. However, their role in BL-induced enhancement of flavonoid synthesis in dandelion remains unclear. We identified 69 WRKY TFs (TmWRKYs) in the complete dandelion genome through rigorous BLAST and HMMER searches, with Arabidopsis WRKY protein sequences and the WRKY domain Pfam model (PF03106) as queries. These proteins were designated TmWRKY1-TmWRKY69 according to chromosomal localization (Table 1, Supplementary Table 3), and their lengths ranged from 153 aa (TmWRKY4) to 1,314 aa (TmWRKY69), corresponding to molecular weights (MW) of 16.66-150.99 kDa. Predicted isoelectric points (pI) spanned 5.0-9.87, with 34 proteins being acidic (pI < 7) and 35 basic (pI > 7). Notably, uniformly negative hydropathicity values confirmed that all are hydrophilic, and all proteins except TmWRKY4 and TmWRKY55 exhibited instability indices > 40, suggesting general instability. Furthermore, subcellular localization predictions indicated that the majority (94.20%) are nuclear; a small subset (4.35%: TmWRKY8, TmWRKY48, TmWRKY51) localized to peroxisomes, while only TmWRKY4 was cytoplasmic (Table 1).

**Table 1:** The physicochemical properties of WRKY family genes in *Taraxacum Mongolicum*

Gene ID	Name	Number of amino acids	MW (kDa)	pI	Instability index	Hydropathicity	Subcellular localization
TbA01G004250.1	TmWRKY1	303	34.27	5.19	70.51	-0.635	nucleus
TbA01G012440.1	TmWRKY2	313	34.91	5.89	68.4	-0.965	nucleus
TbA01G132050.1	TmWRKY3	521	56.82	6.68	48.18	-0.727	nucleus
TbA01G132620.1	TmWRKY4	153	16.66	9.63	28.08	-0.608	cytoplasm
TbA01G139360.1	TmWRKY5	501	55.78	5.71	56.68	-0.844	nucleus
TbA01G150110.2	TmWRKY6	477	53.33	6.58	58.1	-0.962	nucleus
TbA02G001620.1	TmWRKY7	526	57.01	5.77	47.64	-0.633	nucleus
TbA02G034460.2	TmWRKY8	496	55.04	7.36	43.57	-0.988	peroxisomes
TbA02G078350.1	TmWRKY9	272	30.69	7.12	60.03	-0.918	nucleus
TbA02G122210.1	TmWRKY10	355	39.5	9.5	41.02	-0.751	nucleus
TbA02G122540.1	TmWRKY11	228	25.82	7.18	68.21	-0.691	nucleus
TbA02G130450.1	TmWRKY12	337	37.5	6.05	57.84	-0.768	nucleus
TbA02G130580.1	TmWRKY13	265	30.28	9.16	46.22	-0.629	nucleus
TbA02G130630.1	TmWRKY14	304	34.23	6	56.83	-0.656	nucleus
TbA02G133480.1	TmWRKY15	311	33.84	5.65	57.91	-0.727	nucleus
TbA03G128020.1	TmWRKY16	483	53.37	8.66	64.95	-0.809	nucleus
TbA03G121400.1	TmWRKY17	319	34.71	9.56	54.72	-0.57	nucleus
TbA03G096710.1	TmWRKY18	188	21.86	9.62	46.21	-0.891	nucleus
TbA03G065260.4	TmWRKY19	340	37.08	9.69	51.54	-0.626	nucleus
TbA03G036840.2	TmWRKY20	328	36.19	8.37	41.32	-0.734	nucleus
TbA03G025120.2	TmWRKY21	531	57.81	7.72	43.76	-0.63	nucleus
TbA03G019470.1	TmWRKY22	325	36.32	5.41	64.16	-0.978	nucleus
TbA03G013490.1	TmWRKY23	329	36.69	6.31	51.87	-0.979	nucleus
TbA03G004310.1	TmWRKY24	301	32.94	8.28	47.17	-0.759	nucleus
TbA04G096060.1	TmWRKY25	222	25.56	5.37	49.21	-1.04	nucleus
TbA04G092930.1	TmWRKY26	343	38.24	9.68	46.75	-0.808	nucleus
TbA04G088450.1	TmWRKY27	269	30.2	5.49	50.07	-0.883	nucleus
TbA04G082190.1	TmWRKY28	194	22.08	7.69	59.93	-0.822	nucleus
TbA04G079840.1	TmWRKY29	234	27.57	7.65	58.6	-1.352	nucleus
TbA04G078940.1	TmWRKY30	336	38.51	5.87	46.95	-0.78	nucleus
TbA04G077630.2	TmWRKY31	213	24.56	7.62	40.33	-0.863	nucleus
TbA04G071390.1	TmWRKY32	417	45.92	5	52.03	-0.938	nucleus
TbA04G065030.2	TmWRKY33	277	30.94	6.18	58.12	-0.563	nucleus
TbA04G048750.1	TmWRKY34	659	72.1	6.24	55.31	-1.012	nucleus
TbA04G045920.1	TmWRKY35	410	45.88	8.21	44.11	-0.597	nucleus
TbA04G020970.1	TmWRKY36	582	63	6.12	54.71	-0.697	nucleus
TbA04G008700.1	TmWRKY37	248	27.85	8.42	53.88	-1.053	nucleus
TbA05G003680.1	TmWRKY38	347	39.21	9.08	55.32	-0.898	nucleus
TbA05G006020.1	TmWRKY39	274	29.42	9.78	53.64	-0.501	nucleus
TbA05G017010.1	TmWRKY40	335	37.28	9.69	51.68	-0.719	nucleus
TbA05G019340.1	TmWRKY41	293	32.93	6.15	52.11	-0.646	nucleus
TbA05G023360.1	TmWRKY42	491	54.27	6.24	51.7	-0.966	nucleus
TbA05G095610.1	TmWRKY43	496	54.08	7.38	55.75	-0.947	nucleus
TbA05G096680.1	TmWRKY44	297	32.4	6.59	55.33	-0.785	nucleus
TbA05G104990.1	TmWRKY45	337	38.15	5.69	47.97	-0.767	nucleus
TbA05G105360.1	TmWRKY46	218	24.22	5.48	46.44	-0.841	nucleus
TbA06G107970.1	TmWRKY47	350	39.77	5.69	56.05	-0.834	nucleus
TbA06G107950.1	TmWRKY48	211	23.88	8.91	47.08	-0.855	peroxisomes
TbA06G102140.1	TmWRKY49	258	29.2	5.94	41.54	-0.802	nucleus
TbA06G099600.1	TmWRKY50	193	21.9	9.08	41.05	-1.002	nucleus
TbA06G091140.1	TmWRKY51	440	48.59	7.96	51.5	-0.715	peroxisomes
TbA06G070190.1	TmWRKY52	322	36.12	7.1	55.46	-0.666	nucleus
TbA06G001880.1	TmWRKY53	433	48.47	8.39	47.06	-0.443	nucleus
TbA06G001690.2	TmWRKY54	310	34.95	5.23	43.11	-0.573	nucleus
TbA07G001160.1	TmWRKY55	171	18.65	5.05	37.49	-0.829	nucleus
TbA07G007970.1	TmWRKY56	514	56.52	6.22	40.39	-0.64	nucleus
TbA07G013460.1	TmWRKY57	283	31.93	5.39	76.54	-1.026	nucleus
TbA07G017920.1	TmWRKY58	395	43.55	5.92	56.2	-0.843	nucleus
TbA07G019810.1	TmWRKY59	333	36.73	6.17	52.58	-0.911	nucleus
TbA07G028180.1	TmWRKY60	407	45.25	8.23	53.41	-0.856	nucleus
TbA07G031870.1	TmWRKY61	278	30.17	9.87	56.55	-0.582	nucleus
TbA07G036030.1	TmWRKY62	180	20.43	6.59	55.34	-1	nucleus
TbA07G081940.1	TmWRKY63	329	37.14	5.93	50.55	-0.744	nucleus
TbA07G103130.1	TmWRKY64	556	60.62	7.17	47.3	-0.786	nucleus
TbA07G109350.1	TmWRKY65	224	25.67	9.22	51.85	-0.778	nucleus
TbA08G001140.1	TmWRKY66	206	23.85	8.96	56.62	-0.9	nucleus
TbA08G008450.1	TmWRKY67	345	37.29	9.68	46.64	-0.503	nucleus
TbA08G037820.4	TmWRKY68	1314	150.99	6.45	48.79	-0.293	nucleus
TbA08G040560.1	TmWRKY69	223	26.04	7.8	56.55	-1.104	nucleus

### Phylogenetic and amino acid sequence analysis of TmWRKYs

To elucidate evolutionary relationships, a phylogenetic tree was constructed based on multiple alignments of the predicted amino acid sequences of the WRKY domains from dandelion and Arabidopsis using the Neighbor-Joining (NJ) method. Classified according to *Arabidopsis thaliana* (AtWRKYs) groupings, the 69 TmWRKY proteins fall into three main categories: Group I (11 proteins), Group II (47 proteins), and Group III (11 proteins). Group II was further subdivided into five subgroups (IIa–IIe) based on zinc-finger structural motifs, containing 2 (IIa), 9 (IIb), 18 (IIc), 9 (IId), and 9 (IIe) proteins, respectively (Supplementary Fig. 3a). The tree reveals that all clades contain WRKY genes from both species, indicating high evolutionary conservation within this gene family.

Multiple sequence alignment confirmed that the conserved WRKYGQK heptapeptide motif is retained in most TmWRKY proteins (Supplementary Fig. 3b). However, specific deviations were identified in several members. Single amino acid substitutions generated variant motifs: WRKYGKK in TmWRKY31, TmWRKY62, and TmWRKY66; WRKYGLK in TmWRKY68; and WKKYGQK in TmWRKY53. Additionally, double amino acid substitutions resulted in the WKKYGEK motif in TmWRKY39 and TmWRKY61 (Supplementary Fig. 3b).

### Gene structure and conserved motif composition of TmWRKYs

To investigate the structural diversity of the TmWRKY family, exon-intron organization was analyzed using the GSDS tool. All Group I members harbored two WRKY domains, while Group II and III members possessed a single WRKY domain (Supplementary Fig. 4a, b). The 63 TmWRKY genes contained UTRs, and the number of exons ranged from two to six, reflecting significant variation in gene complexity (Supplementary Fig. 4c). MEME motif analysis identified ten conserved motifs among the TmWRKY proteins (Supplementary Fig. 4d). Motif 1, corresponding to the WRKYGQK sequence, was present in almost all members, confirming its evolutionary conservation. Group-specific motif patterns were evident: the members of group IIb contain the largest number of motifs. Motif 4 was exclusively detected in groups I and IIe, and motif 5 was unique in groups IIb and IId, whereas motif 6 was enriched in groups IIa and IIb. *Notably*,* TmWRKY29* and *TmWRKY32* only have the motif 4 (Supplementary Fig. 4d). Such conserved motif combinations may underlie functional specialization among WRKY subgroups.

### Chromosome location, and collinearity analysis of TmWRKYs

The 69 TmWRKY genes in dandelion are distributed across eight chromosomes (Supplementary Table 4). Among them, Chr5 harbors the highest number of TmWRKY genes, with 13 members accounting for 18.8%, followed by Chr8 with 11 TmWRKY genes (15.9%), Chr3, Chr4, and Chr8 each containing 9 TmWRKY genes (13.0%), and Chr7 with 8 TmWRKY genes (11.6%). Chr9 contains the fewest members, with only 4 TmWRKY genes (5.8%) (Supplementary Fig. 5a). Additionally, a tandem duplication event is defined by the presence of two or more genes in close proximity on a chromosome (Supplementary Table 5), and one gene cluster (*TmWRKY47* and *TmWRKY48*) was found on chromosome 7 (Supplementary Fig. 5a). Furthermore, 21 segmental duplication events involved 33 *TmWRKY* genes on chromosomes 2–9, suggesting that specific WRKY genes underwent amplification during dandelion evolution (Supplementary Fig. 5b).

### The prediction of WRKY TFs involved in BL-regulated flavonoid synthesis

**Fig. 5:**
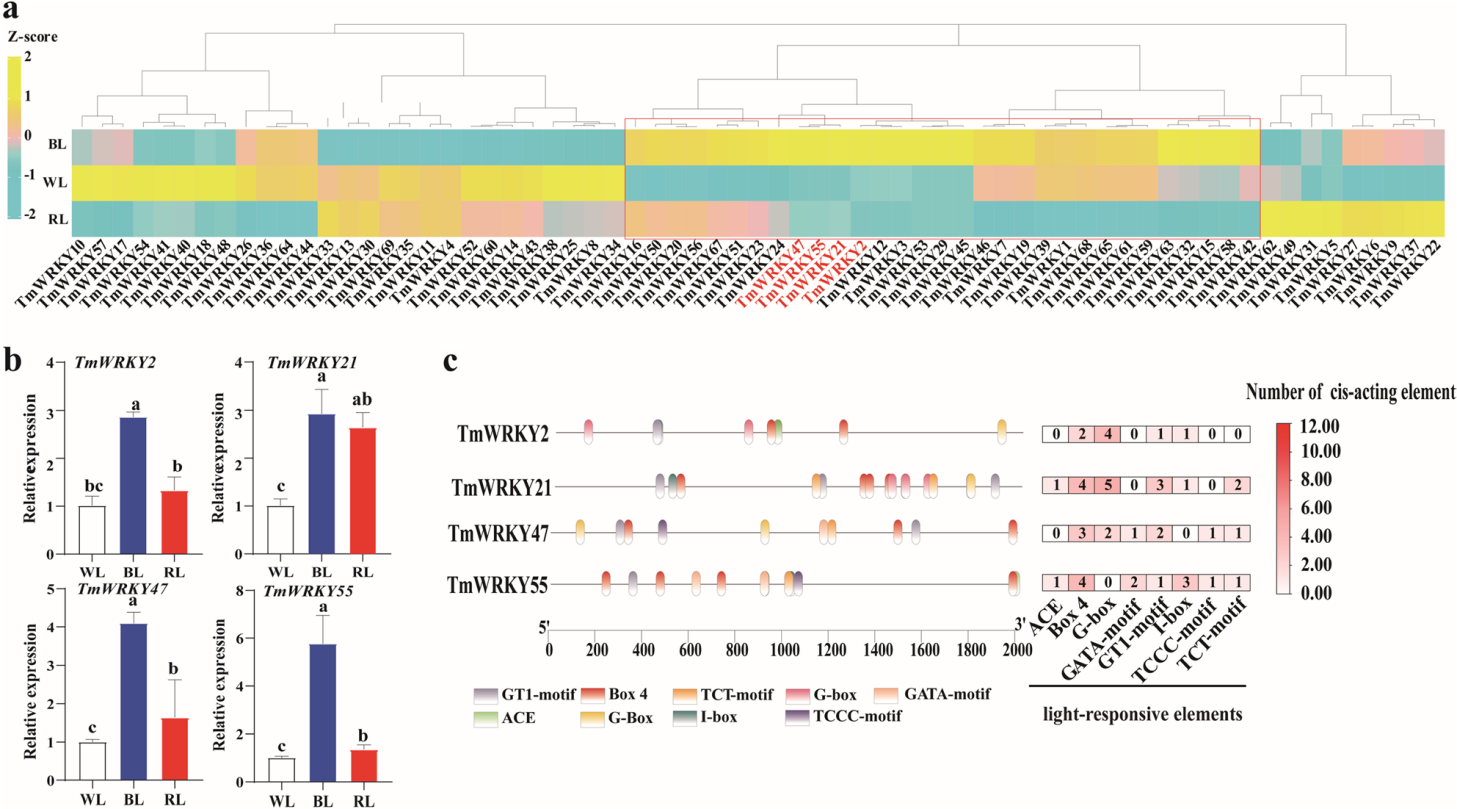
Expression analysis and promoter characterization of BL-induced TmWRKY TFs in dandelion. **a** Heatmaps of the relative expression of *TmWRKYs* under different light treatments. **b** qRT-PCR validation of the expression levels of four *TmWRKY* genes in dandelion leaves under different light treatments. **c** Distribution of functional elements in the promoter region of *TmWRKYs*. Predicted cis-regulatory elements were identified using PlantCARE and visualized with TBtools, where distinct colors represent different cis-acting elements. Values represent as mean ± SD (*n* = 3). Different lowercase letters indicate significant differences at the *P* < 0.05 level. WL, white light; BL, blue light; RL, red light

Transcriptome analysis of dandelion leaves under different light qualities revealed that 31 TmWRKY genes exhibited the highest expression levels in response to BL (Supplementary Table 6). Among these, four genes (*TmWRKY2/21/47/55*) showed significantly higher expression (FDR < 0.05, |log2Fold Change|≥1) under BL compared to under WL and RL (Fig. 5a). qRT-PCR validation confirmed BL-induced up-regulation of these four key *TmWRKY* genes, which was consistent with transcriptomic data (Fig. 5b). To explore the potential mechanism of this up-regulation, we analyzed the promoter regions (2,000 bp upstream of the transcription start site) of these four genes and identified an abundance of light-responsive elements, including ACE-motif, Box 4, G-box, GATA-motif, GT1-motif, I-box, TCCC-motif, and TCT-motif (Fig. 5c). Notably, all four promoters contained both Box 4 and GT1 motifs. In addition to these common elements, *TmWRKY2*, *TmWRKY21*, and *TmWRKY55* also contained I-box elements, while *TmWRKY21*, *TmWRKY47*, and *TmWRKY55* uniquely possessed a TCT motif (Fig. 5c). Collectively, these results indicate that BL induces the transcriptional activation of *TmWRKY2*, *TmWRKY21*, *TmWRKY47*, and *TmWRKY55*.

### Correlation and functional prediction between TmWRKYs and key enzyme genes involved in the flavonoid biosynthesis

**Fig. 6:**
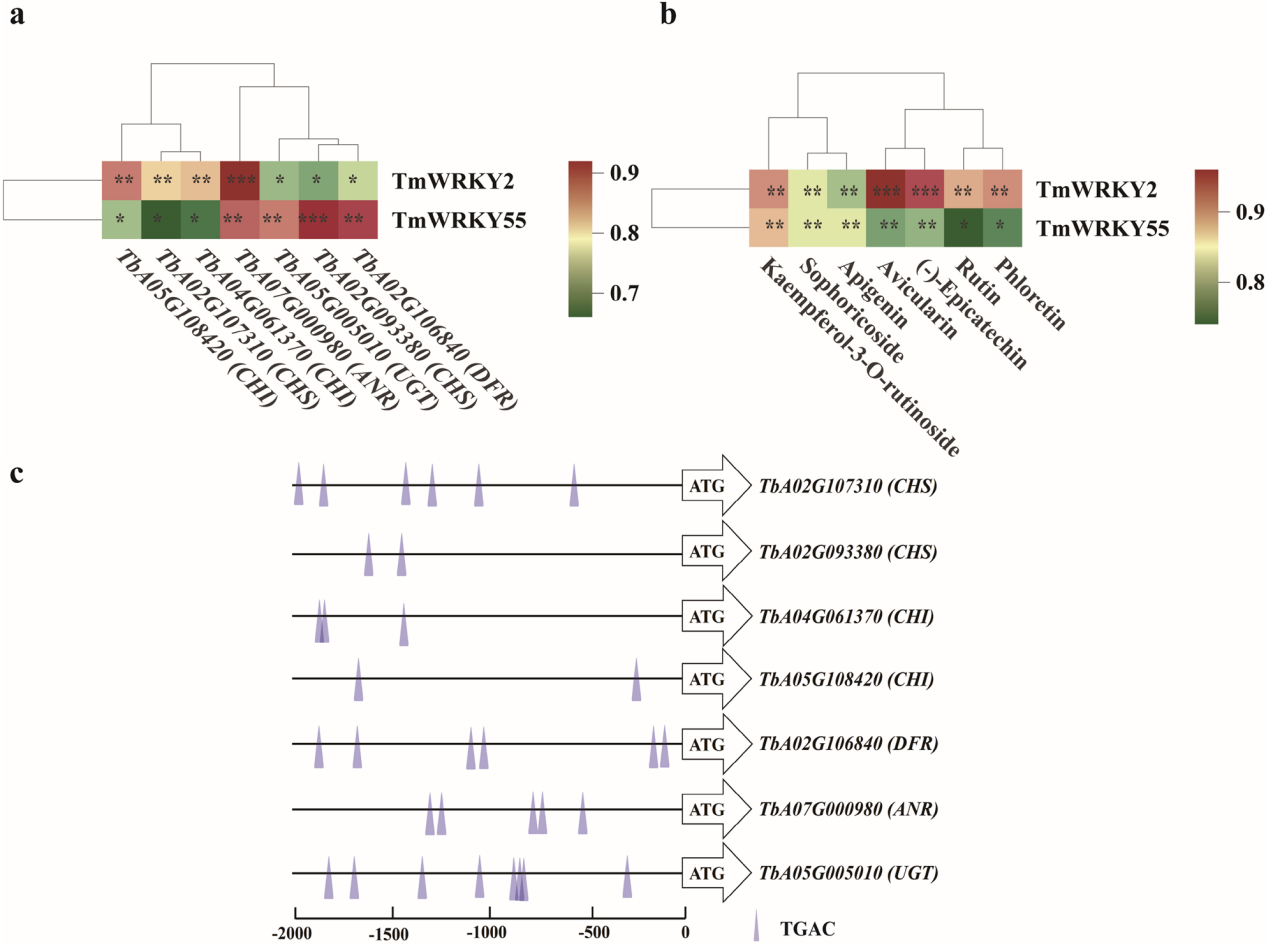
Correlation analyses of *TmWRKYs* and biosynthesis genes involved in antioxidant flavonoids biosynthesis. **a** Correlation analysis of *TmWRKYs* and flavonoid biosynthesis genes in dandelion under a threshold of 0.8 was performed. **b** Correlation analysis of *TmWRKYs* and antioxidant flavonoids in dandelion under a threshold of 0.8 was performed. **c** Schematic diagram of promoter regions of flavonoid biosynthesis structural genes containing WRKY-binding W-box elements. _*_ Indicates significant differences at the *P* < 0.05 level; _**_ indicate significant differences at the *P* < 0.01 level; _***_ indicate significant differences at the *P* < 0.001 level

To explore the potential regulatory role of TmWRKY TFs in the light quality-mediated biosynthesis of antioxidant flavonoids in dandelion, we analyzed the correlations between the expression of four *TmWRKYs* and 9 flavonoid biosynthesis genes, as well as the content of seven antioxidant flavonoids identified through metabolomic screening. The results showed that two *TmWRKYs* exhibited a strong correlation (|r| > 0.8; *P* < 0.01, *P* < 0.05, or *P* < 0.001) with seven key structural genes (Fig. 6a). Specifically, *TmWRKY2* positively correlated with *CHS*,* CHI*,* and ANR*, while *TmWRKY55* showed a significant positive correlation with *CHS*,* DFR*,* ANR and UGT* (Fig. 6a). Furthermore, *TmWRKY2* was positively correlated with the accumulation of all seven antioxidant flavonoids, and *TmWRKY55* was positively correlated with the levels of (-)-epicatechin, apigenin, sophoricoside, and kaempferol-3-O-rutinoside (Fig. 6b). In addition, promoter analysis of the seven correlated structural genes identified many of the core W-box element (TGAC), which suggests that they may be direct targets of the WRKY TFs (Fig. 6c).

## Discussion

Light quality serves as a pivotal environmental signal that modulates plant growth, morphological construction and secondary metabolite [42]. The antioxidant properties of dandelion, largely attributed to its flavonoids, underpin its wide application in medicine and functional foods [14]. Our study establishes BL as a key regulator in dandelion, which concurrently suppresses vegetative growth while potently enhancing the biosynthesis of flavonoids and the associated antioxidant capacity (Figs. 1 and 2). This BL-elicited enhancement of flavonoid accumulation and antioxidant activity aligns with findings in other species, such as basil [23], mugwort [24], sweet pepper [25], rhodiola [26], and cabbage [27]. Furthermore, our integrated multi-omics analysis demonstrated that the expression patterns of specific TmWRKY TFs induced by BL are strongly correlated with the activation of flavonoid biosynthetic pathways (Fig. 6a), suggesting their potential key regulatory role in this process.

Our findings demonstrate that BL is a potent inducer of antioxidant flavonoid biosynthesis (Fig. 3b) by upregulating key structural genes in the pathway (Fig. 4a). Notably, we identified TmWRKY2 and TmWRKY55 as BL-responsive TFs strongly correlated with these metabolic changes (Fig. 6a, b). The presence of conserved W-box motifs in the promoters of these structural genes (Fig. 6c), combined with the strong correlation evidence, suggests a plausible mechanism​ whereby these TmWRKYs directly activate​ the flavonoid biosynthetic pathway in response to BL. This potential mechanism aligns with findings in other species, such as the activation of flavonoid biosynthesis by GhWRKY41 in cotton [43] and FaWRKY71 in strawberry [44]. However, unlike​ the well-characterized direct targets of WRKYs, our study currently provides correlative evidence for TmWRKY2/55. This distinction highlights the need for​ further experimental validation, such as EMSA, LUC, or ChIP-qPCR, to confirm direct promoter binding. Interestingly, while most reported WRKYs act as positive regulators, the repressor function of VvWRKY70 in grape [39] underscores the functional diversity within this protein family and suggests that​ the regulatory effect of TmWRKYs could be specific to the species and BL context.

The presence of conserved light-responsive *cis*-elements in the promoters of *TmWRKY* genes provides crucial clues to their potential roles in BL signaling. Our analysis revealed that all four BL-responsive *TmWRKY* genes harbor Box 4 and GT1-motifs, with subsets additionally containing I-box, TCT, G-box, or ACE elements (Fig. 5c). This specific combination suggests a complex regulatory code for fine-tuning BL responses. The functional importance of such motifs is well-established; for instance, the GT1 motif in the *GhMYC* TF is critical for light-responsive signaling [45], and site-directed mutagenesis has confirmed that the GT1 motif in the *SmPAL1* promoter, along with I-box, ATC-motif, and TCT-motif in *Sm4CL1*, directly mediates responsiveness to blue-red light co-treatment [42]. This conservation strongly implies that the identified motifs are not merely incidental but are likely functional components enabling the BL-induced expression of the *TmWRKY* genes. Notably, the presence of G-box and ACGT core elements is particularly important, as these are the binding sites for HY5, a key TF in the light signaling pathway [46, 47]. Given that BL induces *TmHY5* expression, and TmCOP1-TmHY5 module-mediated BL signal promotes chicoric acid biosynthesis in dandelion [48], we propose a model wherein HY5, activated by BL, may directly bind to the promoters of these *TmWRKY* genes, initiating a transcriptional cascade that ultimately regulates the structural genes of the flavonoid biosynthesis pathway. This hypothesis, however, requires further experimental validation.

While our results clearly demonstrate that TmWRKY21 and TmWRKY47 are transcriptionally activated by BL (Fig. 5a, b), their lack of strong correlation with the key structural genes of the flavonoid pathway (Fig. 6a) suggests a minimal direct role in regulating this secondary metabolic process. Intriguingly, we observed a distinct BL-induced compact phenotype, characterized by shortened petioles and enhanced photosynthetic apparatus development (Fig. 1), which aligns with classic photomorphogenic responses.​ Although the transcript levels of BL photoreceptor genes, such as cryptochromes (cry), showed no significant changes across light treatments (Supplementary Table 8), this pattern suggests that post-translational activation of CRY, rather than transcriptional regulation, serves as the primary trigger for the BL signal transduction cascade [49]. It is well-established that activated CRYs initiate downstream signaling by interacting with COP1/SPA complexes and TF like HY5, ultimately inhibiting cell elongation [50]. This is also supported by the documented role of AtWRKY36-which, like TmWRKY21, belongs to the WRKY IIb subfamily-as a negative regulator of photomorphogenesis [51]. We therefore propose that TmWRKY21, and potentially TmWRKY47, function in a CRY-signaling module to fine-tune morphological adaptations to BL. A primary goal of subsequent research is to test whether TmWRKY21 and TmWRKY47 are integral components of the CRY-mediated signaling cascade that shapes BL-induced morphological changes.

**Fig. 7:**
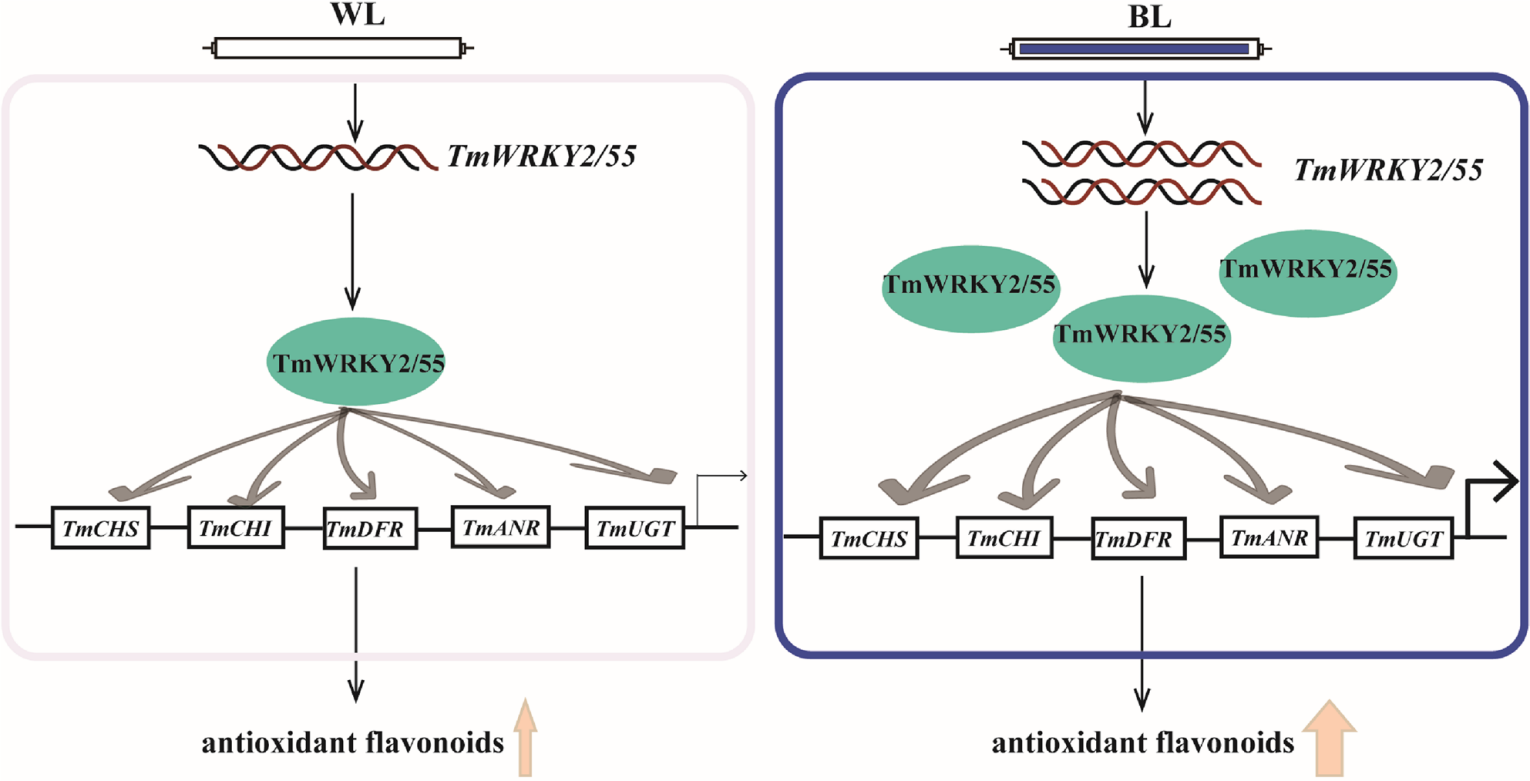
The proposed model of TmWRKYs involvement in BL-induced antioxidant flavonoid synthesis. Potential WRKY target genes in the flavonoid biosynthesis pathway are shown in rectangles, and WRKY proteins are represented by ellipses. The thickness of the arrows indicates the strength of their effects, and wavy lines represent WRKY transcripts

In summary, this study establishes the first genome-wide characterization of the WRKY family in *Taraxacum mongolicum*, integrating multi-omics evidence to understand how blue light enhances flavonoid accumulation in dandelion via transcriptional activation. We propose a model in which BL signals are transduced through specific TmWRKY TFs-particularly TmWRKY2 and TmWRKY55-to upregulate the expression of key enzyme genes in the flavonoid pathway, thereby increasing the production of antioxidant metabolites and strengthening the plant’s medicinal properties (Fig. 7). These findings not only advance our mechanistic understanding of light-regulated secondary metabolism in non-model medicinal plants but also offer practical targets for molecular breeding and light-quality-assisted cultivation of dandelion with improved nutraceutical value.

## Materials and methods

### Plant growth and different light treatments

As previously reported [52], dandelion (*Taraxacum antungense*) biomaterials were collected, transplanted in the greenhouse of our laboratory. The seeds were sown in a solid substrate mixture of peat, vermiculite, and perlite (2:1:1, v/v/v). Germination was conducted in a growth chamber, and then the 60-day-old dandelion seedlings with uniform and well-grown morphology were selected and randomly assigned to three groups. These groups were subsequently exposed to BL, RL, and full-spectrum WL (as the control) LED light treatments for a period of 36 days, respectively. The light intensity for all light quality treatments was adjusted to and maintained at 200 µmol m⁻² s⁻¹ using the spectrometer (ASENSE TEK). The photoperiod was 16/8 h (light/dark) for all groups and the relative humidity was 60%. Three biological replicates of five plants per treatment group were set up. Some of the samples were soaked in liquid nitrogen and stored at -80 ℃ for liquid chromatography tandem mass spectrometry (LC-MS) and RNA-seq analysis as well as qRT-PCR analysis. Some of the samples were dried in an oven (55 ℃) until constant weight, ground, sieved, and used for flavonoid content analysis.

### Measurement of pigments

The pigment content was determined as described by He et al. [53]. Briefly, fresh leaf samples (0.1 g) were cut into pieces and immersed in 10 mL of 95% ethanol in a test tube. All tubes were then placed in darkness at room temperature for 48 h. After extraction, the solution was mixed thoroughly, and a portion was transferred to a cuvette. Absorbance was measured at wavelengths of 470, 649, and 665 nm using a UV spectrophotometer, with 95% ethanol serving as the blank.

### Measurement of antioxidant activities

The DPPH and ABTS^+^ free radical-scavenging activity was evaluated according to the method described previously [54]. Ferric reducing antioxidant power (FRAP) assay was s determined using a previously reported method [55].

### Genome-wide identification and physicochemical properties analysis of WRKY genes in dandelion

The whole genome and protein sequences of dandelion plants (*Taraxacum mongolicum*) were obtained from the National Genomics Data Center (https://ngdc.cncb.ac.cn/search). The reported AtWRKY sequences were downloaded from the TAIR database (www.arabidopsis.org). The conserved WRKY domain sequence (PF03106; HMM) was gained from the Pfam database (https://www.pfam.sanger.ac.uk/). The potential TmWRKY proteins in the dandelion genome were identified by the BLASTp method and TBtools software using the HMMER3.0 program [56], followed by de-redundancy analysis. Subsequently, the SMART tool (http://smart.embl-heidelberg.de/) and the CDD (https://www.ncbi.nlm.nih.gov/cdd) database were applied for domain identification. The physicochemical properties and subcellular localization predictions of the identified TmWRKY family members were implemented using ProtParam (https://web.expasy.org/protparam/) and WoLF PSORT (https://psort.hgc.jp/), respectively.

### Phylogenetic tree, motif composition, and gene structure analysis of TmWRKY TFs

Multiple sequence alignment of WRKY family proteins from *Taraxacum mongolicum* and *Arabidopsis thaliana* was performed using MEGA 7.0 on the basis of ClustalW default parameters. A phylogenetic tree was subsequently constructed via the Neighbor-joining method and visually optimized using iTOL (https://itol.embl.de). Conserved motifs of the TmWRKY protein sequences were analyzed using MEME software (https://meme-suite.org/meme/tools/meme). Both gene structure and conserved domains are finally visualized using TBtools software [56].

### Chromosomal distribution, collinearity analysis, and cis-acting elements analysis of TmWRKY genes

Chromosomal localization and collinearity analysis of the TmWRKY genes were displayed using the TBtools software [56] based on the *Taraxacum mongolicum* genome annotation file obtained from the National Genomics Data Center. Cis-acting elements in the 2000 bp upstream promoter sequences of TmWRKY genes were identified using the PlantCARE platform (http://bioinformatics.psb.ugent.be/webtools/plantcare/html/), followed by graphical representation with TBtools software [56].

### Total flavonoid analysis

Total flavonoid content was determined using the Flavonoid Assay Kit BC1330 (Solarbio, Beijing, China) according to the manufacturer’s instructions. Briefly, fresh samples were dried to constant weight and ultrasonically extracted in a 60 °C water bath (300 Hz) for 30 min. The extracts were then centrifuged (12,000 rpm, 10 min, 25 °C). Absorbance of the mixture was measured at 470 nm using a microplate reader. A standard curve was constructed using rutin reference standard (98% purity, BC1330, Solarbio, Beijing, China) at a series of concentrations. Total flavonoid content in dandelion was quantified based on the standard curve.

### Metabolomics analysis

Dandelion leaves that had been treated with WL, RL, and BL for 36 days were used for metabolite extraction using an 80% methanol (v/v) solution with ultrasonic assistance, respectively. Widely-argeted metabolomics analysis was performed by Novogene Co., Ltd. (Beijing, China) using ultra-high-performance liquid chromatography coupled with tandem mass spectrometry. The LC-MS/MS system was established at Novogene Co. In addition, three quality control samples were prepared by mixing aliquots of each experimental sample. The chromatographic conditions mainly included the mobile phase: aqueous phase A (with 0.1% formic acid added) and acetonitrile (with 0.1% formic acid added) for phase B. The elution gradient: 2% B from 0 to 2 min, 2% B to 100% B from 2 to 15 min, 100% B from 15 to 17 min, 100% B to 2% B from 17 to 17.1 min, 2% B from 17.1 to 20 min at a flow rate of 0.4 mL/min and a column temperature of 50 degrees. Pairing an ExionLC AD system (SCIEX) with a QTRAP 6500 + mass spectrometer (SCIEX). Mass spectrometry conditions: Curtain Gas: 35 psi; Collision Gas: Medium; IonSpray Voltage: -4500 V; Temperature: 550℃; Ion Source Gas 1: 60; Ion Source Gas 2: 60. The mass spectral information obtained was compared with novoDB (novogene database) to identify the metabolites qualitatively. The samples were detected in multiple reaction monitoring (MRM) mode, and the mass spectra were opened by SCIEX OSV1.4 software to obtain the qualitative and quantitative results of the metabolites.

### Transcriptome analysis

Total RNA was isolated from 3 biological replicates of dandelion leaves subjected to 36 d treatments of WL, RL, and BL, respectively, by ethanol precipitation and CTAB-PBIOZOL. Subsequently, RNA concentration and integrity were assessed using a Qubit fluorescence quantifier and a Qsep400 high-throughput biofragment analyzer. After passing the library check, the different libraries were sequenced on the Illumina platform. Data quality control was performed using fastp to remove reads with adapters, and HISAT was used to build an index, and clean reads were aligned to the reference genome of *Taraxacum mongolicum* (GWHBCHG00000000). Gene expression levels were quantified using FPKM (Fragments Per Kilobase Million). Differentially expressed genes (DEGs) were defined as those meeting the threshold of fold change ≥ 1.5 with false discovery rate (FDR)-adjusted “p-value < 0.05”, as determined by DESeq2.

### High performance liquid chromatography (HPLC) analysis

For rutin quantification, separation was performed on a Phenomenex Luna C18 column (250 × 4.6 mm, 5 μm) at 30 °C. The mobile phase comprised methanol (A) and 0.1% phosphoric acid in water (B) with the following gradient: 13% A to 20% A from 0 to 7 min, 20% A to 30% A from 7 to 18 min, 30% A to 41% A from 18 to 28 min, 41% A to 45% A from 28 to 35 min, 45% A to 62% A from 35 to 38 min, 62% A to 69% A from 38 to 45 min, and 69% A to 95% A from 45 to 50 min. The flow rate was 1.0 mL min⁻¹, the detection wavelength 254 nm, and the injection volume 10 µL. For quercetin and apigenin analysis, the same column and temperature were used with acetonitrile (A) and 0.1% formic acid in water (B). Gradient: 5% A to 10% A from 0 to 7 min, 10% A to 25% A from 7 to 25 min, 25% A to 30% A from 25 to 30 min, and 30% A to 40% A from 30 to 37 min. Other parameters are identical to rutin analysis. Calibration curves demonstrated excellent linearity over the tested concentration ranges: Quercetin: y = 9 × 10^7^x−530.88.9 (R^2^ = 0.996), Rutin: y = 4 × 10^7^x−88,227 (R^2^ = 0.998), Apigenin: y = 6 × 10^7^x+706.69 (R^2^ = 0.999).

### Quantitative real-time PCR (qRT-PCR) analysis

Frozen dandelion leaves (0.1 g) that had been treated with different light conditions were pulverized in liquid nitrogen using a pre-chilled mortar. Total RNA was extracted with the RNAprep Pure Plant Kit (DP432, TIANGEN) following the manufacturer’s instructions, with RNA integrity verified by 1.5% agarose gel electrophoresis. And then reverse transcribe to cDNA according to the manufacturer’s instructions of HiScript III All-in-one RT SuperMix Perfect for qPCR (Vazyme, Nanjing, China). The qRT-PCR is performed using the ChamQ SYBR qPCR Master Mix (Vazyme, Nanjing, China), according to the instructions. The relative gene expression was calculated using the Eq. 2^−ΔΔCt^, and the gene-specific primers (synthesized by Sangon Biotech, Shanghai) with sequences provided in Supplementary Table 7 were employed for amplification.

### Statistical analysis

Statistical analyses were performed using SPSS 21.0 (IBM Corp.). One-way ANOVA with LSD test at *P* < 0.05 significance level was applied to determine significant differences among experimental groups. Data visualization was conducted in GraphPad Prism 8.0, generating bar graphs with error bars representing mean ± SD (*n* = 3 biological replicates).

## Supplementary Information

Supplementary Material 1.

Supplementary Material 2.

## Data Availability

The RNA-seq data have been deposited in the National Genomics Data Center database (https://ngdc.cncb.ac.cn/gsa) under accession number PRJCA050236. All other data supporting the findings of this study are available within the paper and its Supplementary Information.
