# Supplementary material for: Blue light as an eco-elicitor effectively enhances flavonoid biosynthesis and antioxidant capacity in dandelion revealed by integrated metabolomic and transcriptomic analyses
Source: BMC Plant Biol. 2026 Jan 19;26:286. doi: 10.1186/s12870-026-08137-1 (PMC12895943; doi:10.1186/s12870-026-08137-1)
Supplement: Supplementary file 2 — Supplementary Material 2. [file 12870_2026_8137_MOESM2_ESM.docx]

Supplementary Fig. 1 Differential metabolite profiling in dandelion leaves under different light treatments **a** Principal component analysis (PCA) of LC-MS/MS results in dandelion leaves treated with different light qualities. **b** Correlation analysis between all metabolomic samples. **c** Primary metabolite distribution profiles under varied light wavelengths. The KEGG enrichment analysis of DAMs in BL vs WL (**d**) and BL vs RL (**e**) comparison group. WL, white light; BL, blue light; RL, red light.


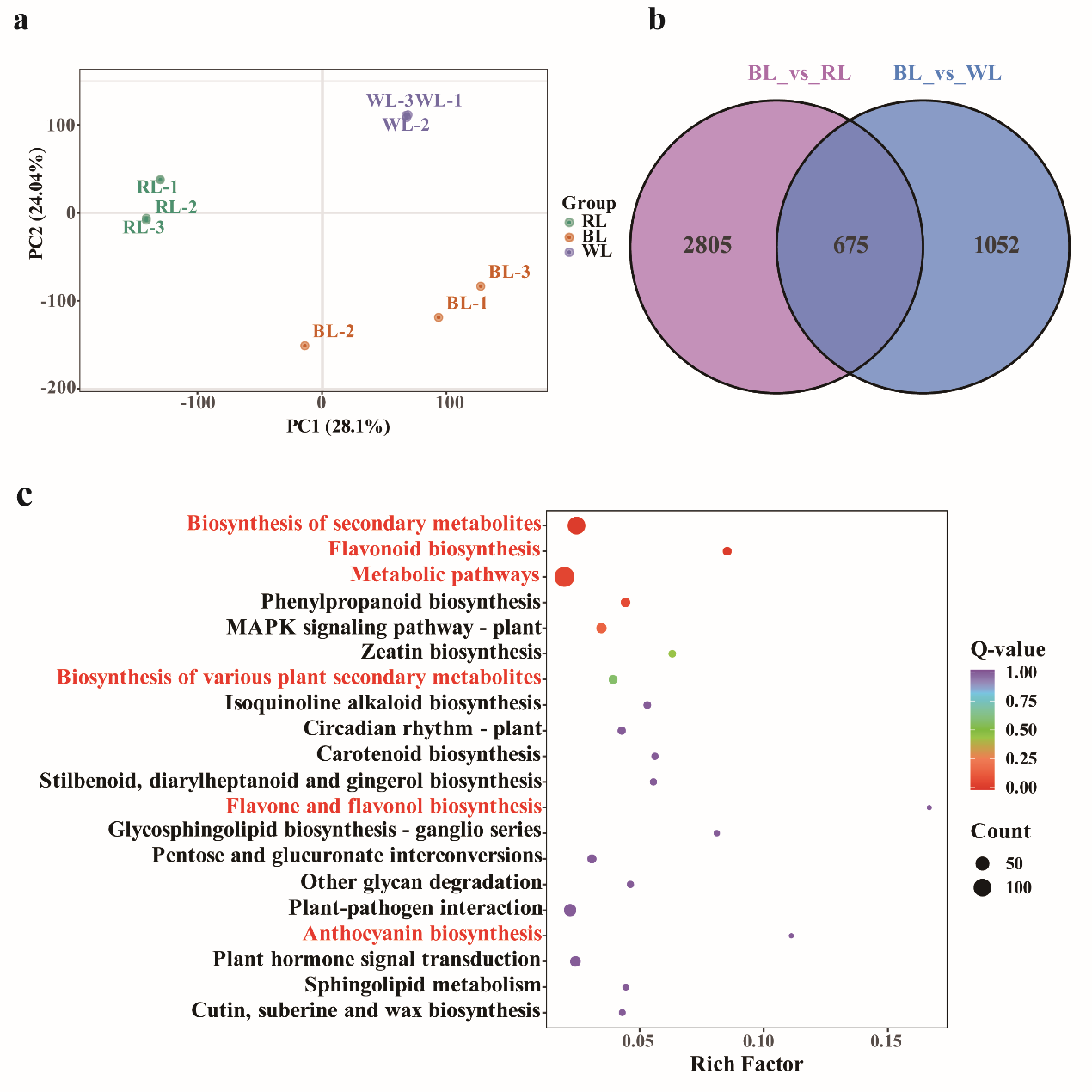


Supplementary Fig. 2 Differential transcriptomic profiling in dandelion leaves under different light treatments **a** Principal component analysis (PCA) of RNA-seq results in dandelion leaves treated with different light qualities. **b** Venn diagram of DEGs for different comparison groups (BL vs RL and BL vs WL). **c** KEGG enrichment analysis of DEGs common to both the BL vs RL and BL vs WL comparisons. WL, white light; BL, blue light; RL, red light.


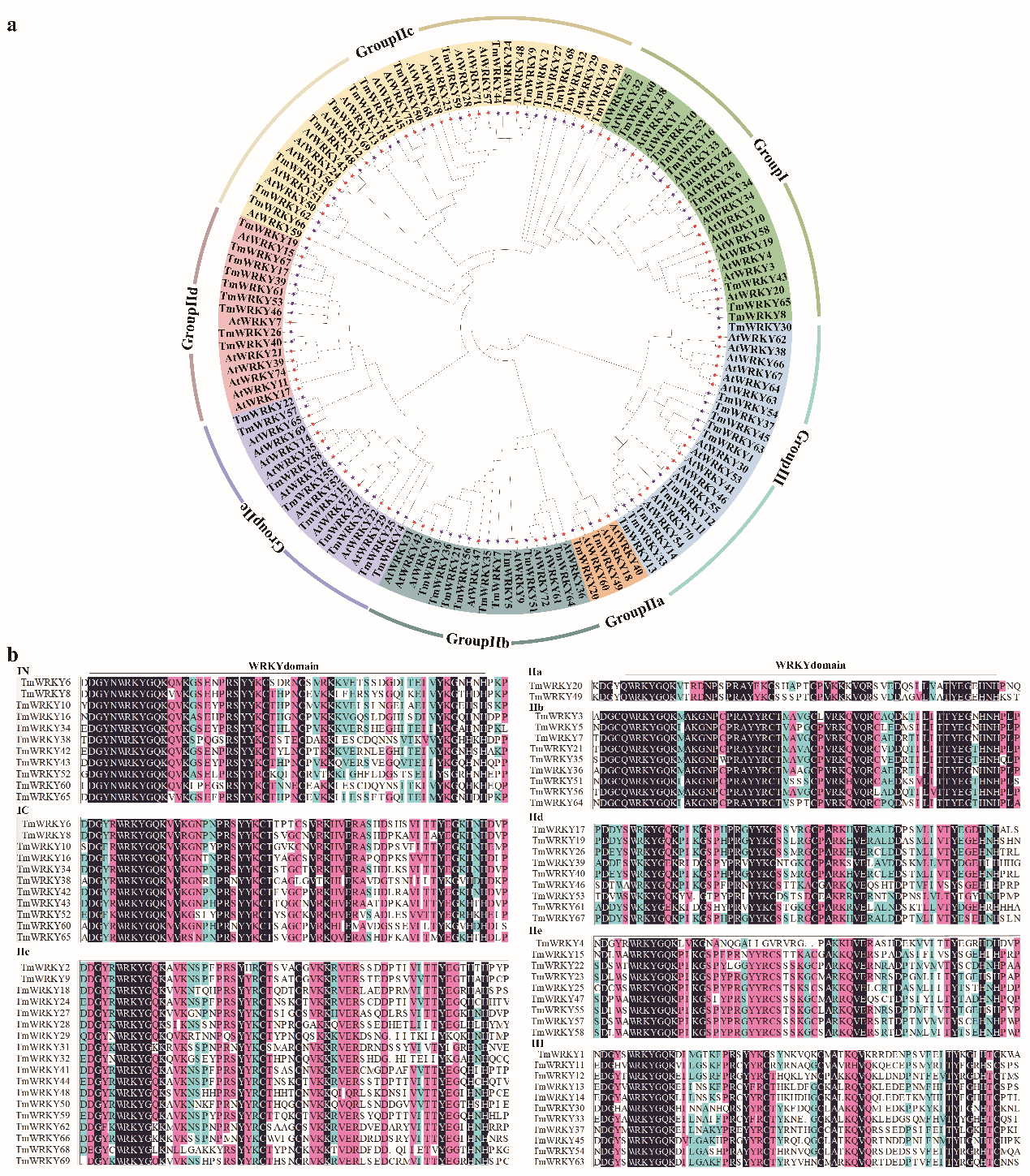


Supplementary Fig. 3 Phylogenetic and WRKY protein domain sequence analysis of *Taraxacum mongolicum*. **a** Phylogenetic relationships of the TmWRKY gene family among *Taraxacum mongolicum*, *Arabidopsis*, and lettuce plants. Protein sequences were aligned using ClustalW, followed by phylogenetic tree construction in MEGA 11.0 employing the Neighbor-Joining (NJ) method with 1000 bootstrap replicates. **b** WRKY protein domain sequence analysis in *Taraxacum mongolicum*.


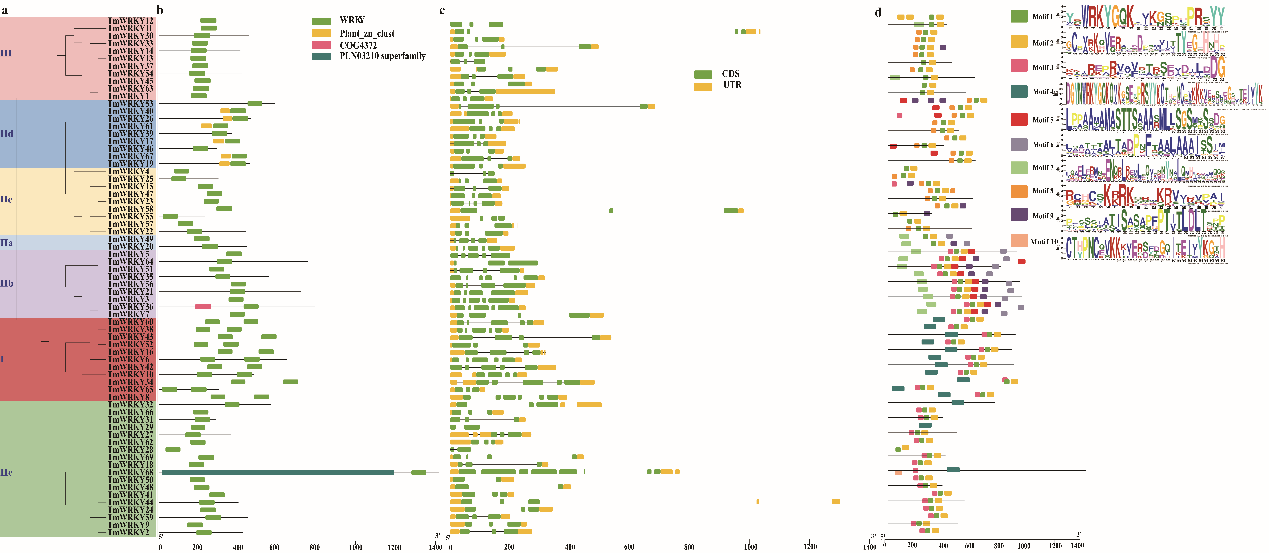


Supplementary Fig. 4 The evolutionary relationship, genetic structures, and conserved motifs of TmWRKYs. **a** Phylogenetic evolution of TmWRKY genes. **b** Conserved domain analysis of TmWRKYs. **c** Gene structure visualization of the TmWRKY gene family in *Taraxacum mongolicum*. **d** Conserved motifs distribution of WRKY protein of *Taraxacum mongolicum*.


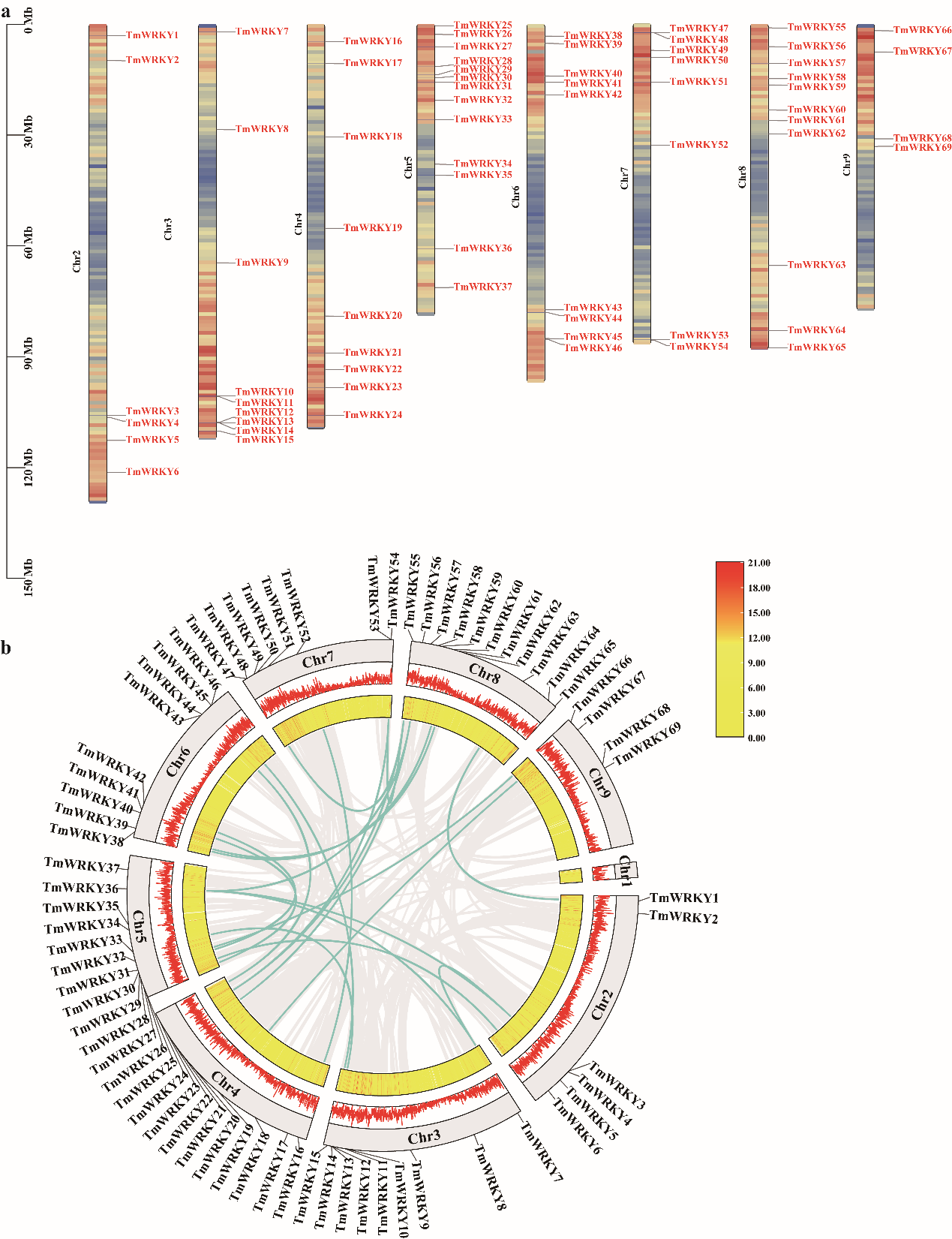


Supplementary Fig. 5 Chromosomal localization and collinearity analysis of TmWRKYs. **a** Chromosomal localization diagram of WRKY genes. **b** Internal synteny circle diagram of the *Taraxacum mongolicum* genome. Blue lines indicate collinear *TmWRKY* gene pairs.
